# Supplementary material for: Resolving the full spectrum of human genome variation using Linked-Reads
Source: Genome Res. 2019 Apr;29(4):635–45. doi: 10.1101/gr.234443.118 (PMC6442396; doi:10.1101/gr.234443.118)
Supplement: Supplemental Material [file supp_gr.234443.118_Supplemental_longranger-2.2.zip › longranger-2.2.tar.bz2!/lr2.2-public/lib/stan/cmdstan/stan/doc/api/html/argument_8hpp_source.html]

Stan: src/stan/gm/arguments/argument.hpp Source File


|  |  |
| --- | --- |
|  | Stan  2.5.0  probability, sampling & optimization |


- Main Page
- Related Pages
- Namespaces
- Classes
- Files

- File List
- File Members

All Classes Namespaces Files Functions Variables Typedefs Enumerations Enumerator Friends Macros Pages

- src
- stan
- gm
- arguments

argument.hpp

Go to the documentation of this file.

1 #ifndef STAN\_\_GM\_\_ARGUMENTS\_\_ARGUMENT\_\_BETA

2 #define STAN\_\_GM\_\_ARGUMENTS\_\_ARGUMENT\_\_BETA

3

4 #include <vector>

5 #include <string>

6 #include <sstream>

7 #include <iomanip>

8

9 namespace stan {

10  namespace gm {

11

12  class argument {

13  public:

14

15  argument()

16  : indent\_width(2),

17  help\_width(20) { }

18

19  argument(const std::string name)

20  : \_name(name),

21  indent\_width(2),

22  help\_width(20) { }

23

24  virtual ~argument() { }

25

26  std::string name() const {

27  return \_name;

28  }

29

30  std::string description() const {

31  return \_description;

32  }

33

34  virtual void print(std::ostream\* s, const int depth, const std::string prefix) = 0;

35  virtual void print\_help(std::ostream\* s, const int depth, const bool recurse) = 0;

36

37  virtual bool parse\_args(std::vector<std::string>& args,

38  std::ostream\* out,

39  std::ostream\* err,

40  bool& help\_flag) {

41  return true;

42  }

43

44  virtual void probe\_args(argument\* base\_arg, std::stringstream& s) {};

45

46  virtual void find\_arg(std::string name,

47  std::string prefix,

48  std::vector<std::string>& valid\_paths) {

49  if (name == \_name) {

50  valid\_paths.push\_back(prefix + \_name);

51  }

52  }

53

54  static void split\_arg(const std::string& arg, std::string& name, std::string& value) {

55  size\_t pos = arg.find('=');

56

57  if (pos != std::string::npos) {

58  name = arg.substr(0, pos);

59  value = arg.substr(pos + 1, arg.size() - pos);

60  }

61  else {

62  name = arg;

63  value = "";

64  }

65  }

66

67  virtual argument\* arg(const std::string name) {

68  return 0;

69  }

70

71  int compute\_indent(const int depth) {

72  return indent\_width \* depth + 1;

73  }

74

75  protected:

76  std::string \_name;

77  std::string \_description;

78

79  int indent\_width;

80  int help\_width;

81  };

82

83  } // gm

84 } // stan

85 #endif

86

stan::gm::argument::probe\_args

virtual void probe\_args(argument \*base\_arg, std::stringstream &s)

**Definition:** argument.hpp:44

stan::gm::argument::argument

argument()

**Definition:** argument.hpp:15

stan::gm::argument::description

std::string description() const

**Definition:** argument.hpp:30

stan::gm::argument::argument

argument(const std::string name)

**Definition:** argument.hpp:19

stan::gm::argument::find\_arg

virtual void find\_arg(std::string name, std::string prefix, std::vector< std::string > &valid\_paths)

**Definition:** argument.hpp:46

stan::gm::argument::split\_arg

static void split\_arg(const std::string &arg, std::string &name, std::string &value)

**Definition:** argument.hpp:54

stan::gm::argument::compute\_indent

int compute\_indent(const int depth)

**Definition:** argument.hpp:71

stan::gm::argument::help\_width

int help\_width

**Definition:** argument.hpp:80

stan::gm::argument::~argument

virtual ~argument()

**Definition:** argument.hpp:24

stan::gm::argument::\_name

std::string \_name

**Definition:** argument.hpp:76

stan::gm::argument::indent\_width

int indent\_width

**Definition:** argument.hpp:79

stan::gm::argument::parse\_args

virtual bool parse\_args(std::vector< std::string > &args, std::ostream \*out, std::ostream \*err, bool &help\_flag)

**Definition:** argument.hpp:37

stan::gm::argument::print\_help

virtual void print\_help(std::ostream \*s, const int depth, const bool recurse)=0

stan::gm::argument::name

std::string name() const

**Definition:** argument.hpp:26

stan::gm::argument::print

virtual void print(std::ostream \*s, const int depth, const std::string prefix)=0

stan::gm::argument::arg

virtual argument \* arg(const std::string name)

**Definition:** argument.hpp:67

stan::gm::argument

**Definition:** argument.hpp:12

stan::gm::argument::\_description

std::string \_description

**Definition:** argument.hpp:77


---

[ Stan Home Page ]

*© 2011–2014,
Stan Development Team.*
